# Rumen Bacteria Communities and Performances of Fattening Lambs with a Lower or Greater Subacute Ruminal Acidosis Risk

**DOI:** 10.3389/fmicb.2017.02506

**Published:** 2017-12-12

**Authors:** Fei Li, Zhilan Wang, Chunxiao Dong, Fadi Li, Weimin Wang, Zehu Yuan, Futao Mo, Xiuxiu Weng

**Affiliations:** ^1^State Key Laboratory of Pastoral Agricultural Ecosystem, Key Laboratory of Grassland Livestock Industry Innovation, Ministry of Agriculture, College of Pastoral Agriculture Science and Technology, Lanzhou University, Lanzhou, China; ^2^Engineering Laboratory of Sheep Breeding and Reproduction Biotechnology in Gansu Province, Minqin, China; ^3^College of Animal Science and Technology, Gansu Agricultural University, Lanzhou, China

**Keywords:** rumen acidosis, cellulolytic bacteria, bacteria diversity, biomarker, sheep

## Abstract

Several ruminal cellulolytic bacteria species are sensitive to pH and could therefore be used as biomarkers to determine the risk of sub-acute ruminal acidosis (SARA) in finishing lambs. This study compared a 2–4 h post feeding ruminal pH measurement to abundances of the ruminal pH-sensitive bacteria to evaluate the risk of SARA in a herd of 120 finishing lambs. The lambs were reared in individual units for 50 days. Ruminal fluid was collected by use of an orogastric tube on day 51 2-4 h after feeding. Although the lambs were fed an identical diet, they responded differently in the abundances of four ruminal pH sensitive cellulolytic bacteria (*Ruminococcus albus, Ruminococcus flavefaciens* and *Fibrobacter succinogenes* and *Butyrivibrio fibrisolvens*). Lambs with the most or the least cellulolytic bacteria were then classified as either lower SARA risk (LSR, *n* = 10) or higher SARA risk group (HSR, *n* = 10), respectively. Data showed that the ruminal pH and VFA profiles were uncorrelated with the number of cellulolytic bacteria (*P* > 0.050). Lambs with the HSR showed lower ruminal pH (*P* = 0.013) and acetate to propionate ratio (*P* = 0.018), higher concentrations of lactate (*P* = 0.035) and proportion of propionate (*P* = 0.033) compared to those with the LSR. The DMI and ADG did not differ in LSR and HSR lambs (*P* > 0.050). A diversity analysis revealed significantly lower diversity in HSR lambs than in LSR (Simpson index, *P =* 0.004). The relative abundances of the phyla *Bacteroidetes, Fibrobacteres, Verruomicrobia*, and *Proteobacteria* were higher in LSR lambs than in HSR (*P* < 0.050). The abundances of several phyla including *Firmicutes, Tenericutes* and *Actinobacteria* were higher in the HSR than in the LSR group (*P* < 0.050). The bacterial communities of the LSR and HSR clustered separately in rumen based on the Unifrac distances, indicating distinct bacteria communities at OTU level between the LSR and HSR lambs. Overall, there was no correlation between 2 and 4 h post-feeding ruminal pH and the abundance of pH-sensitive bacteria and the amount of these bacteria could be used as a potential biomarker of SARA in lamb herd.

## Introduction

Highly fermentable diets are often used with high-producing ruminants, especially during the finishing period for meat sheep or the peak milking period for dairy cows (Plaizier et al., 2008). However, excessively fermentable diets can reduce ruminal pH and increased the risk of subacute ruminal acidosis (SARA). This disease affects rumen function, and can cause milk fat depression, rumen epithelium damage and laminitis in cattle, leading to unnecessary economic losses (Kleen and Cannizzo, 2012). Several nutritional strategies have been proposed to minimized the incidence of SARA, including recommend minimum dietary forage NDF (National Research Council, 2001) or physically effective NDF (peNDF) levels (Zebeli et al., 2010); however, some individuals in a herd develop SARA regardless. Previous studies reported that significant variations in the severity of SARA among sheep, beef cattle and dairy cows occur despite the animals being fed identical diets (Gao and Oba, 2016). Subacute ruminal acidosis did not often present overt clinical symptoms and the clinical signs are usually delayed from the time of incidence (Colman et al., 2015). Therefore, it is necessary to identify biomarkers to diagnosis individual SARA risks in herds to aid early prevention of SARA in individuals and herds.

Ruminal pH profiles have been extensively used to determine the incidence of SARA on ruminant individuals and herds. The time spent or the area below the pH threshold (pH × min) when ruminal pH below 5.80 or 5.60 during a 24 h period, which were proposed as metrics by Penner et al. (2009), have been widely used to evaluate individual SARA severity on sheep (Penner et al., 2009), beef steer (Schlau et al., 2012) and lactating dairy cow (Gao and Oba, 2016) in clinical trials. In field trials, however, the one time (2–8 h after feeding) ruminal fluid pH is used to reflect herd SARA prevalence (pH < 5.5), and ruminal fluid is usually obtained by rumenocentesis or use of an orogastric tube (Kleen and Cannizzo, 2012). Duffield et al. (2004) found that the ruminal pH varied greatly at times between 0 and 6 h after feeding indicating that one-time pH measurements do not exactly reflect diurnal ruminal pH profiles and the risk of SARA (Penner et al., 2006). A system that continuously monitors pH values is desirable to assess rumen health, but this system cannot be widely adopted in herd trials because it is expensive and owing to animals usually lacking rumen fitulas and difficult to recycle the devices (Steiner et al., 2015). Therefore, alternative methods or biomarkers must be developed to determine the risk of SARA at the herd level. Cellulolytic bacteria including *Ruminococcus albus, Ruminococcus flavefaciens, Fibrobacter succinogenes* and *Butyrivibrio fibrisolvens* have been suggested to be sensitive to pH (Nagaraja and Titgemeyer, 2007). Studies on dairy cows, sheep and beef steers have shown that the number of cellulolytic bacteria decreased when animals experienced grain-induced SARA (Li et al., 2014; Plaizier et al., 2017), which indicated that the change in the abundance of these bacteria can reflect the dynamic ruminal pH. In addition, Li et al. (2009) also found that although sampling time affects pH, bacterial diversity and predominant cellulolytic bacteria species (*F. succinogenes, R. albus, R. flavefaciens*, and *B. fibrisolvens*) in bovine rumen do not changed. These results indicated bacteria communities are thus more stable over time than one-time point pH measurements. We therefore hypothesize that predominant ruminal cellulolytic bacteria can be used to assess individual SARA risks in lamb herd.

The first objective of the present experiment was to investigate the correlation between the 2–4 h post-feeding ruminal pH and the abundance of pH-sensitive bacteria in ruminal fluid. Another objective was to study the validity of using pH-sensitive bacteria to differentiate SARA risk of individuals in lamb herd that were fed identical diets.

## Materials and Methods

This experiment was approved by the Biological Studies Animal Care and Use Committee of Gansu Province, China (2005–12) and conducted according to their established guidelines

### Animal and Diets

This study was conducted with 120 male *Hu* lambs with similar body weight and birth age. After the lambs adapted to the experiment diets for 10 days, they were weighted at 0600-0800 (before morning feeding). The lambs were reared in individual units (0.75 m × 1.5 m × 1.0 m) so that their dry matter intake (DMI) and other performance parameters could be measured for 50 days. The feed was prepared in the form of a total mixed ration (TMR) pellet that contained 27% barely straw and 73% concentrate (Supplementary Table S1) and was offered at 5% of body weight as feed bases at the start of the experiments; then the feed was supplied at 110% of actual feed intake from the previous day. All animals were fed three times per day at 0700, 1500, and 1900 h and allowed free access to water. The amounts of feed offered and orts were recorded daily throughout the experiment.

### Sample Collection and Analysis

The lambs were weighted on two consecutive days after they were reared in individual units for 50 days. Then, ruminal fluid was collected from each lamb by use of an orogastric tube 2–4 h after morning feeding. The ruminal fluid was mixed thoroughly and filtered through 4 layers of cheesecloth.

The ruminal fluid pH was immediately determined using a pH meter. An 8 mL aliquot of ruminal fluid was preserved with adding 1 mL of metaphosphoric acid (25% wt/vol) to determine volatile fatty acid (VFA) content. The rest of the samples were stored at -20°C for bacteria DNA extraction. For VFA determination, thawed samples of the rumen fluid were centrifuged for 15 min at 10,000 × *g* at 4°C. Two milliliters of the supernatant were then mixed with 200 μL crotonic acid (1% wt/vol), and the solution was filtered through a 0.45 μm filter. The ruminal VFAs were separated and quantified by using a gas chromatograph (Trace 1300, Thermo Fisher Scientific, United States) as described by Li et al. (2014), using a 30 m × 0.32 mm × 0.33 μm fused silica column (DB-FFAP, Agilent Technologies, United States). Lactate concentrations in the ruminal fluid were determined using commercially available lactate assay kits (A019-2, Nanjing Jiancheng Bioengineering Institute, Nanjing, China).

### DNA Isolation and Quantitative PCR Analysis of Bacterial NDA

Ruminal fluid was thawed and mixed, and the total genomic DNA was extracted from samples of approximately 200 μL by using an E.Z.N.A. Stool DNA kit (Omega Bio-Tek, Norcross, GA, United States), following the manufacturer’s instructions. The obtained bacterial DNA samples were used as templates in quantitative real-time PCR (qRT-PCR). The primers that were used in qRT-PCR are listed in Supplementary Table S2. Real-time PCR amplification was performed in triplicate using a Bio-Rad CFX96 Real-Time System (Bio-Rad Laboratories, Hercules, CA, United States). The reaction was run in a final volume of 20 μL in 96-well plates; the reaction consisted of 10 μL of SYBR Green (TransGen Biotech, Beijing, China), 1 μL of bacterial DNA, 0.2 μL of each primer, and 8.6 μL of ddH_2_O. All bacterial DNA was amplified using the following program: 94°C for 1 min and then 40 cycles of denaturing at 94°C (5 s), annealing at 60°C (15 s), and 72°C for 10 s consisted of one cycle at 94°C and 42 denaturing cycles at 94°C. Absolute quantification for all bacteria using specific 16S rDNA standards. The results for counting of each species were expressed as log10 copy number of 16S rRNA gene copies per mL rumen fluid. The total number of cellulolytic species was calculated as the sum of numbers of *R. albus, R flavefaciens, F. succinogenes* and *B. fibrisolvens*. Twenty lambs’ rumen bacterial DNA were chosen for 16S rRNA gene sequencing, and they were evenly taken from represented the lower SARA risk lambs (LSR, with the most cellulolytic bacteria) and the higher SARA risk lambs (HSR, with least cellulolytic bacteria).

### 16S rRNA Gene Sequencing and Bioinformatics Analysis

Bacteria DNA from LSR (*n* = 10) and HSR (*n* = 10) lambs was used for 16S rRNA gene sequencing and bioinformatics analysis. Bacterial universal primers 5′-ACTCCTACGGGAGGCAGCA-3′, 5′-GGACTACHVGGGTWTCTAAT-3′ to amplify the V3-V4 region of the bacterial 16S rRNA gene and subsequently sequence the PCR products. The PCR amplicons were sequenced by using an Illumina HiSeq 2500 platform at Biomarker Technologies Co. Ltd. (Beijing, China). Overlapping paired-end reads of the DNA fragments (minimum overlap of 10 bp, maximum mismatch rate set 0.2) were merged by using FLASH v1.2.7^1^ (Bolger et al., 2014). Low-quality sequences were trimmed using a minimum average quality score of 20, with a window size of 50 bp. Chimeric reads were filtered using UCHIME v4.2 software^2^, and reads from each sample were clustered into Operational Taxonomic Units (OTUs) based on 97% sequence similarity according to the UCLUST in QIIME (Caporaso et al., 2010; Edgar, 2010). Representative sequences of each OTU were assigned a taxonomy by RDP Classifier and then aligned against entries in the Silva alignment database (Release 128) (Yilmaz et al., 2014). Samples were rarefied for alpha diversity calculations, including generating curves of their OTU rank, rarefaction and Shannon index. Standard Shannon and Simpson indices and richness indices including the Ace and Chao1 index were determined by using Mothur (version v.1.30). To compare the dissimilarities in bacterial communities in LSR and HSR lambs, we performed a principal coordinate analysis (PCoA) that was based on weighted UniFrac distances at OTU level. The sequencing data were deposited into the Sequence Read Archive (SRA) of NCBI and can be accessed via accession number SRP108931.

### Statistical Analysis

Individual lamb rumen fermentation parameters, and bacteria abundances were analyzed using the PROC CORR procedure of SAS to analyze the correlation between parameters (version 9.2; SAS Institute Inc., Cary, NC, United States). The UNIVARIATE procedure was used to test the normality of the residuals. Abnormally distributed data were log transformed as described by McCann et al. (2016). The PROC TTEST procedure was used to analyze the differences between results from HSR and LSR lambs, including their performances, rumen fermentation parameters and bacteria abundances. Significance was declared at *P* < 0.05, and trends were declared at 0.05 < *P* < 0.10.

## Results

The four ruminal cellulolytic bacteria (*F. succinogenes, R. flavefaciens, R. albus* and *B. fibrisolvens*) ranged from 9.22 to 12.76 log_10_ 16S rRNA copies/mL rumen fluid among all lambs. The lambs with the lowest (*n* = 10) and highest (*n* = 10) number of cellulolytic bacteria were categorized as HSR and LSR, respectively. The correlation between the number of cellulolytic bacteria and the fermentation parameters displayed in **Table 1**. The four cellulolytic bacterium abundances positively correlated with each other (*P* < 0.01, *r* > 0.400), but had no relationship with ruminal pH and VFA concentrations. The abundance of *B. fibrisolvens* had a moderate positive correlation with the acetate to propionate ratio (*P* = 0.044, *r* = 0.189). The ruminal pH was negatively correlated with total VFA (*P* < 0.01, *r* = -0.249) and the propionate molar ratio (*P* < 0.01, *r* = -0.301) but positively correlated with the acetate molar ratio (*P* < 0.01, *r* = 0.323) and acetate to propionate ratio (*P* < 0.01, *r* = 0.368).

**Table 1:** Correlation analysis of cellulolytic bacteria and rumen fermentation parameters (*n* = 120).

Items^1^	Fs.	Rf.	Ra.	Bf.	Tc.	pH	TVFA	Ac.	Pr.	Bu.	Ac:Pr
Fs.	1										
Rf.	0.413**	1									
Ra.	0.473**	0.537**	1								
Bf.	0.588**	0.520**	0.401**	1							
Tc.	0.723**	0.768**	0.661**	0.877**	1						
pH	0.106	0.172	0.018	0.042	0.101	1					
TVFA	–0.032	–0.157	–0.082	–0.034	–0.093	–0.249**	1				
Ac.	0.073	0.113	0.123	0.146	0.148	0.323**	–0.149	1			
Pr.	–0.048	–0.076	–0.111	–0.121	–0.117	–0.301**	0.141	–0.848**	1		
Bu.	0.001	–0.077	–0.036	–0.017	–0.041	0.026	0.020	0.119	–0.607**	1	
Ac:Pr	0.141	0.053	0.113	0.189*	0.176	0.368**	–0.166	0.850**	–0.963**	0.534^∗∗^	1

*^1^Fs., *Fibrobacter succinogenes*; Rf., *Ruminococcus flavefaciens*; Ra., *Ruminococcus albus*; Bf., *Butyrivibrio fibrisolvens*; Tc, Total cellulolytic bacteria; TVFA, Total volatile fatty acid; Ac., Acetate; Pr., Propionate; Bu., Buytrate*

The animal performance and rumen fermentation parameters measured in 120 lambs are illustrated in **Table 2**. The lambs in the LSR and HSR groups had similar initial body weight (*P* = 0.531), final body weight (*P* = 0.849), DMI (*P* = 0.824), average daily growth (ADG, *P* = 0.391) and DMI to ADG ratio (*P* = 0.523). The ruminal pH (*P* = 0.013), acetate molar ratio (*P* = 0.001) and acetate to propionate ratio (*P* = 0.018) were significantly higher in LSR lambs than in HSR lambs. Concentration of lactate in LSR was lower than the HSR lambs (*P* = 0.035).

**Table 2:** Performance and rumen fermentation parameters in lower SARA risk (LSR) and higher SARA risk (HSR) lambs.

Items	LSR	HSR	Pooled SEM	P-value
Initial BW, kg	22.16	23.03	0.67	0.531
Final BW, kg	35.06	35.38	0.80	0.849
DMI, kg/d	1.19	1.18	0.03	0.824
ADG, kg/d	0.258	0.247	0.01	0.391
DMI:ADG	4.61	4.78	0.12	0.523
Ruminal pH	6.55	6.17	0.08	0.013
Total VFA, m*M*	71.80	73.58	5.68	0.880
**VFA molar ratios, mol/100 mol**				
Acetate	62.10	58.70	0.58	0.001
Propionate	25.17	29.36	1.00	0.033
Isobutyrate	0.89	0.67	0.07	0.114
Butyrate	9.82	9.58	0.65	0.860
Isovalerate	1.00	0.74	0.09	0.153
Valerate	1.02	0.94	0.04	0.405
Acetate:propionate	2.48	2.00	0.10	0.018
Lactate, m*M*	0.54	1.07	0.12	0.035
**Bacteria, Log_10_ 16S rRNA copy number/mL rumen fluid**				
Total bacteria	16.08	15.38	0.21	0.108
Total cellulolytic bacteria	12.35	10.33	0.24	<0.001
%in total bacteria × 10^-3^	40.35	6.29	10.24	0.008
*Fibrobacter succinogenes*	11.12	9.54	0.21	<0.001
*Ruminococcus flavefaciens*	11.83	9.23	0.32	<0.001
*Ruminococcus albus*	11.10	9.22	0.25	<0.001
*Butyrivibrio fibrisolvens*	11.87	9.98	0.24	<0.001
*Prevotella brevis*	10.26	9.57	0.17	0.132
*Streptococcus bovis*	7.92	7.33	0.11	0.030
*Selenomonas ruminantium*	12.38	11.52	0.13	<0.001

We measured the four cellulolytic bacterium abundances in high rumen pH (*n* = 10) and low rumen pH lambs (*n* = 10) and found no significant differences (*P* > 0.05, **Table 3**) between the abundances of the two group. The total bacterial abundance was not significantly different in the ruminal fluid of LSR and HSR lambs (*P* = 0.108, **Table 4**). However, the number of 16S rRNA gene copies of the four cellulolytic bacteria were greater in LSR lambs than in HSR.

**Table 3:** Rumen pH and bacteria abundances in lambs with high-pH and low-pH

Items	High rumen pH lambs	Low rumen pH lambs	Pooled SEM	*P*-value
Ruminal pH	6.83	5.75	0.13	<0.001
Bacteria, Log_10_ 16S rRNA copies number/mL rumen fluid				
Total bacteria	15.64	15.42	0.10	0.311
Total cellulolytic bacteria	11.69	11.42	0.11	0.254
*Fibrobacter succinogenes*	10.79	10.53	0.11	0.418
*Ruminococcus flavefaciens*	10.84	10.28	0.22	0.311
*Ruminococcus albus*	10.77	10.63	0.11	0.254
*Butyrivibrio fibrisolvens*	11.20	10.96	0.14	0.331

**Table 4:** Alpha-diversity and relative abundances of dominant phyla in lower SARA risk (LSR) and higher SARA risk (HSR) lambs.

Phylum	LSR	HSR	Pooled SEM	P-value
**Alpha-diversity**				
OTU	1140.5	1142.6	23.0	0.965
ACE	1214.2	1218.1	21.8	0.931
Chao1	1232.3	1246.4	23.4	0.772
Shannon	5.48	5.42	0.04	0.455
Simpson	0.0112	0.0156	0.0008	0.004
Coverage, %	99.74	99.78	0.02	0.341
**Phylum, %**				
*Bacteroidetes*	43.58	34.71	1.35	<0.001
*Firmicutes*	41.97	54.24	1.67	<0.001
*Fibrobacteres*	3.87	0.99	0.52	0.007
*Verrucomicrobia*	3.37	1.87	0.31	0.011
*Spirochaetes*	2.21	1.99	0.18	0.569
*Proteobacteria*	2.02	0.69	0.24	0.005
*Tenericutes*	1.13	3.10	0.31	0.001
*Lentisphaerae*	0.29	0.70	0.07	0.003
*Saccharibacteria*	0.50	0.12	0.07	0.007
*Actinobacteria*	0.47	1.30	0.14	0.001
*Elusimicrobia*	0.26	0.09	0.04	0.063
*Cyanobacteria*	0.11	0.04	0.04	0.391
*Synergistetes*	0.10	0.05	0.01	0.064
*SR1 (Absconditabacteria)*	0.08	0.03	0.01	0.007
*Chloroflexi*	0.03	0.07	0.01	0.088
*Armatimonadetes*	0.01	0.01	0.00	0.533
*Firmicutes* to *Bacteroidetes* ratio	0.97	1.60	0.09	<0.001

### Diversity of Ruminal Bacterial Communities

The 16S rRNA sequencing of ruminal bacteria DNA samples from 20 lambs clustered into LSR and HSR groups produced 1,363,215 sequence reads after filtering for quality, averaging at 68,161 reads per sample. The average read length was 456 bp (ranging from 454 to 459). In total, we detected 28 phyla, 30 classes, 43 orders, 70 families and 182 genera. At phyla level, *Firmicutes* and *Bacteroidetes* were the most abundant, accounting for 87.25% of all sequences (relative abundances of 48.1 and 39.15%, respectively). The relative abundances of five phyla beside *Firmicutes* and *Bacteroidetes* higher than 1%: *Fibrobacteres* (2.43%), *Spirochaetae* (2.10%), *Verrucomicrobia* (2.62%), *Proteobacteria* (1.35%), and *Tenericutes* (2.11%). The abundance of the *Bacteroidetes* (43.58 vs. 34.71%, *P* < 0.001), *Fibrobacteres* (3.87 vs. 0.99%, *P* = 0.007) and *Absconditabacteria* (0.08 vs. 0.03%, *P* = 0.007) phyla were higher in the LSR group than in the HSR group (**Table 4**). However, the HSR group had a greater abundance of *Firmicutes* (54.24 vs. 41.97% *P* < 0.001), *Tenericutes* (3.10 vs. 1.73%, *P* = 0.001), and *Actinobacteria* (1.30 vs. 0.47%, *P* = 0.010) than the LSR group. The abundances of *Spirochaetes* (2.21 vs. 1.99%, *P* = 0.569), *Elusimicrobia* (0.26 vs. 0.09%, *P* = 0.063), *Cyanobacteria* (0.11 vs. 0.04%, *P* = 0.391) and *Chloroflexi* (0.03 vs. 0.07%, *P* = 0.088) did not differ between the LSR and HSR groups.

At the genus level, we have only listed the bacterial genera with greater than 1% relative abundance (**Table 5**). We found that the relative abundances of 13 of the 24 bacterial genera with >1% relative abundance significantly differed between LSR and HSR lambs including *Prevotellaceae* UCG-001 (1.32 vs. 0.70%, *P* = 0.001), *Saccharofermentans* (2.98 vs. 5.27%, *P* = 0.007), *Ruminococcus* 1 (2.27 vs. 1.11%, *P* = 0.001), *Ruminococcaceae* UCG-014 (0.99 vs. 2.41%, P = 0.004), *Ruminococcaceae* UCG-010 (1.81 vs. 1.19%, *P* = 0.033), *Ruminococcaceae* NK4A214_group (3.50 vs. 5.90%, *P* = 0.026), *Succiniclasticum* (3.76 vs. 1.91%, *P* = 0.001), *Lachnospiraceae* AC2044 group (2.05 vs. 1.23%, *P* = 0.043), *Christensenellaceae* R-7 group (6.05 vs. 12.40%, *P* < 0.001) and *Fibrobacter* (3.87 vs. 0.99%, *P* = 0.007). Several unclassified bacteria genera with greater than 1% relative abundance belongs to the families of *Bacteroidales* S24-7 group, *Bacteroidales* BS11 gut group and *Lachnospiraceae*. *Bacteroidales* S24-7 group (8.36 vs. 4.83%, *P* = 0.017) and *Acidaminococcaceae* (3.76 vs. 1.91%, *P* = 0.001) family abundances were higher in LSR lambs than HSR lambs, while *Ruminococcaceae* (15.79 vs. 21.54%, *P* < 0.001) and *Christensenellaceae* (6.05 vs. 12.40%, *P* < 0.001) abundances were lower in LSR lambs than in HSR lambs. The abundances of *Rikenellaceae* RC9 gut group (9.16 vs. 8.00%, *P* = 0.283), and *Prevotella* 1 (10.01 vs. 10.46%, *P* = 0.743) were not significantly different in the LSR and HSR lambs.

**Table 5:** Relative abundances of dominant families and genera lower SARA risk (LSR) and higher SARA risk (HSR) lambs (%).

Family	Genus	LSR	HSR	Pooled SEM	P-value
*Rikenellaceae*	*Rikenellaceae RC9_gut_group*	9.16	8.00	0.53	0.283
*Prevotellaceae*		13.45	12.32	0.68	0.424
	*Prevotellaceae UCG-001*	1.32	0.70	0.11	0.001
	*Prevotella 1*	10.01	10.46	0.66	0.743
*Bacteroidales_S24-7_group*		8.36	4.83	0.75	0.017
	*Rumen bacterium*	4.28	2.10	0.43	0.006
	*Bacterium*	4.08	2.73	0.43	0.128
*Bacteroidales_BS11_gut_group*		9.65	8.00	0.80	0.318
	*Rumen bacterium*	4.62	4.87	0.76	0.873
	*Bacterium*	5.03	3.13	0.62	0.127
*Ruminococcaceae*		15.79	21.54	0.89	<0.001
	*Saccharofermentans*	2.98	5.27	0.43	0.007
	*Ruminococcus 1*	2.27	1.11	0.20	0.001
	*Ruminococcaceae UCG-014*	0.99	2.41	0.25	0.004
	*Ruminococcaceae UCG-010*	1.81	1.19	0.15	0.033
	*Ruminococcaceae NK4A214 group*	3.50	5.90	0.53	0.026
*Lachnospiraceae*		11.63	11.74	0.46	0.903
	*Lachnospiraceae AC2044 group*	2.05	1.23	0.21	0.043
	*Lachnoclostridium 10*	1.31	0.93	0.26	0.471
	*Bacterium*	2.01	1.94	0.15	0.832
*Christensenellaceae*	*Christensenellaceae R-7_group*	6.05	12.40	0.85	<0.001
*Acidaminococcaceae*	*Succiniclasticum*	3.76	1.91	0.31	0.001
*Fibrobacteraceae*	*Fibrobacter*	3.87	0.99	0.52	0.007
*Spirochaetaceae*	*Treponema 2*	2.01	1.76	0.18	0.508

**FIGURE 1:**
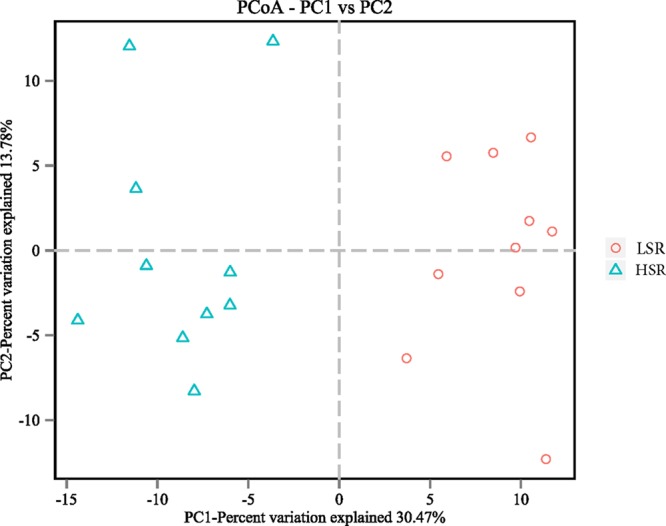
Two dimensional PCoA plots based on weighted UniFrac distance.

Bacterial richness and diversity parameters are reported in **Table 4**. The OTU number and ACE, Chao1 and Shannon indices were not significantly different in LSR and HSR lambs (*P* > 0.05), while the Simpson index in LSR lambs was lower than that in HSR (0.011 vs. 0.016, *P* = 0.004). The OTU β-diversity was determined by using the phylogeny-based UniFrac method. The results of a PCoA analysis of rumen bacterium were depicted in **Figure 1**. This analysis indicated that the OTUs of HSR and LSR lambs were separated into two clusters with weighted UniFrac distances.

## Discussion

In the present study, we found that 2–4 h post feeding ruminal pH was uncorrelated with the amount of pH sensitive bacteria, such as *F. succinogenes, R. flavefaciens* and *R. albus*. And the amount of the four cellulolytic bacteria were similar in lambs with low and high pH (**Table 4**), indicating that a lower 2–4 h post ruminal pH may not be a suitable indicator of SARA risk. Ruminal pH values obtained from 2 to 4 h post feeding have been widely used to evaluate SARA risk in dairy herds, but none of these studies examined correlations between the ruminal pH and bacterial abundances (Morgante et al., 2007; Kleen et al., 2009). A decreased abundance in ruminal cellulolytic bacteria is a typical symptom of SARA, because the number of these bacteria is decreased when the ruminal pH is less than 6.0 (Nagaraja and Titgemeyer, 2007). Li et al. (2009) found rumen fluid sampling times relative to feeding (3 h pre-feeding, 3 and 9 h post-feeding) did not affect the abundances of the four cellulolytic bacterial species (*F. succinogenes, Ruminococcus albus, R. flavefaciens* and *B. fibrisolvens*) despite the rumen pH (6.46, 5.95, 5.59, *P* < 0.01) changing over time. As the ruminal pH potentially varies during the day, the one-time pH measurements cannot accurately reflect ruminal pH dynamics. The similarity in pH-sensitive bacterium communities in lambs with high and low ruminal pH indicated that both groups of lambs had similar diurnal pH fluctuations. The results of our study therefore suggested that measuring the 2–4 h post feeding ruminal pH may not be suitable to accurately estimating SARA risk in lambs.

In our study, the abundances of four-pH sensitive bacteria were used as biomarkers to classify SARA risk in lambs. As expected, positive correlations among the four pH sensitive bacteria indicated that the populations of these bacteria responded less than 2–4 h ruminal pH levels to ruminal pH fluctuations. Several experiments on dairy cow and goat have noted that the abundances of pH-sensitive bacteria, including *F. succinogenes, R. flavefaciens, R. albus* and *B. fibrisolvens* were decreased during grain induced SARA (Khafipour et al., 2009; Li et al., 2014; Mickdam et al., 2016). However, these bacteria have not been used as indicators of SARA. Although parameters that describe diurnal pH fluctuations, including the time spent and the area below the pH thresholds (5.80 or 5.60) are better than the average ruminal pH or 2–4 h post feeding pH metrics in reflecting rumen health, the dynamic ruminal pH in HSR and LSR lambs were not determined, and correlations between pH-sensitive bacterium abundances and ruminal pH profiles require further study.

In the present study, risk of SARA was independent of DMI and dietary composition (**Table 2**) and this observation was consistent with those of previous studies (Schlau et al., 2012; Gao and Oba, 2014, 2016) that did not report DMI differences in greater SARA risk and lower SARA risk animals (SARA risk defination based on the area of rumen pH below 5.8 divide by dry matter intake). Similar DMI by LSR and HSR lambs could be attributed to their similar body weight and rumen volume (Nasrollahi et al., 2017). We observed significant differences in ruminal pH between LSR and HSR (6.55 vs. 6.17, *P* = 0.013) lambs, although cellulolytic bacteria species abundance and ruminal pH did not correlate. As LSR and HSR lambs had similar DMI and dietary composition, the difference in ruminal pH cannot be attributed to dietary factors. A decrease in ruminal pH is usually associated with a high concentration of total VFA and lactate (Li et al., 2014). However, the total VFA concentration in LSR and HSR lambs was also similar. In previous studies, Gao and Oba (2016) and Nasrollahi et al. (2017) observed no differences in VFA concentration in LSR and HSR groups in mid-lactation dairy cows, while Schlau et al. (2012) observed different VFA content in LSR and HSR steers. The lower ruminal pH in HSR lambs, compared with LSR lambs, was likely due to the presence of a higher concentration of lactate.

There were no significant differences in DMI, ADG and feed efficiency between in the LSR and HSR groups. Experimental data relating the risk of SARA on growth performances of finishing lambs or cattle are limited, especially under identical dietary conditions. Schlau et al. (2012) also found no significant differences in DMI between LSR and HSR steers (5.84 vs. 5.63 kg/d, *P* = 0.72); however, they did not determine ADG and feed efficiency. Experiments by Mohammed et al. (2012) and Gao and Oba (2016) indicated that milk yield, milk fat percentage and fat yield of dairy cows were not affected by the severity of SARA. However, an experiment by Rezac et al. (2014) found cattle with severe rumenitis lesions associated with a significant decrease in ADG (0.025 kg/d, *P* < 0.001) and hot carcass weight (-2.20 kg, *P* < 0.001). The relationships between SARA and growth performance of ruminants is still not clear. In our study, we did not find evidence that associated the risk of SARA with the growth performance of lambs when they were fed a common diet.

Rumen bacterial diversity was lower in HSR lambs than LSR lambs (Simpson index, 0.011 vs. 0.002, *P* = 0.001). Similar results were obtained by Fernando et al. (2010) and Mao et al. (2013) when exposing cattle and steer to high concentrate feeds induced SARA. Chen et al. (2012) also found that bacterial diversity was lower in HSR steers than LSR steers by using the PCR-DDGE method. These results indicated that both HSR and high-grain induced SARA could reduce the evenness of microflora. Bacteria community richness did not differ in LSR and HSR lambs in our study, which is consistent with the results of McCann et al. (2016), who found that the bacterial communities of cows with a high risk of SARA and non-SARA cows with identical dietary intake had similar richness. In contrast, Mao et al. (2013) and Wetzels et al. (2017) observed reduced in richness during grain-induced SARA. These differences may be due to the different experimental designs in the studies. Risk of SARA in the present study was independent of dietary factors, while SARA in those studies was induced by increasing dietary grain levels. We speculate that bacterial richness is independent of pH and mainly affected by changes in dietary composition.

Bacterial composition is generally determined by substrate composition (e.g., Cellulose and starch) and extracellular environment (e.g., pH and osmotic pressure) (Nagaraja, 2016). When SARA is induced by increasing dietary grain levels (Mao et al., 2013; Petri et al., 2013), the bacterial community that established in rumen is the combined effect of fermentable substrates and dynamic pH (Because ruminal pH decrease with increasing dietary grain level). Three groups of bacteria communities change under grain-induced SARA: pH-sensitive but substrate insensitive bacteria, pH-insensitive but substrate sensitive bacteria, and bacteria that are both pH- and substrate-sensitive. Therefore, the differences in bacterial communities in the HSR and LSR groups resulted from their different pH levels because both groups were given identical feed composition and exhibited identical DMI.

The UniFrac distance analysis showed that bacterial communities of the HSR and LSR lambs clustered separately, indicating their distinct bacterial compositions in the rumen. Several studies have shown that high-grain induced SARA can affect the diversity and composition of ruminal microflora in cattle (Khafipour et al., 2009; Mao et al., 2013; McCann et al., 2016). Mao et al. (2013) using 454 pyrosequencing analysis showed that the relative abundance of the phylum *Bacteroidetes* decreased during high-concentrate induced (70% concentrate feed, DM basis) SARA. In the present study, the lower relative abundances of *Bacteroidetes* in HSR lambs could be attributed to the low ruminal pH because many gram-negative *Bacteroidetes* are sensitive to pH (Kampmann et al., 2012; Wetzels et al., 2017). The relative abundance of ruminal *Firmicutes* increases with increasing dietary concentrate proportion (Wetzels et al., 2015; Kim et al., 2016), because rapidly fermentable carbohydrates promote *Firmicutes* proliferation (Kallus and Brandt, 2012; Wetzels et al., 2017). In our study, the differences in *Firmicutes* abundances in LSR and HSR lambs are independent of dietary compositions, because both groups had similar DMI and were fed identical diets. An *in vitro* study showed that pH plays a key role in shaping bacterial community compositions and that the low G+C% gram-positive *Firmicutes* dominated when the pH was 5.54, whereas at high pH (pH = 6.71), *Bacteroides* outcompeted them (Duncan et al., 2009; Salonen and de Vos, 2014). Therefore, the higher relative abundance of *Firmicutes* in HSR lambs over LSR lambs could be partly explained by the lower ruminal dynamic pH in the HSR lambs. In the present study, the abundance of *Actinobacteria* was higher in HSR lambs. Studies in calf and dairy cattle also found that the *Actinobacteria* increased with decreasing ruminal pH (Mao et al., 2013; Kim et al., 2016).

*Fibrobacteres* is a critical bacterial phylum that degrades plant cellulose in ruminants (Rosenberg, 2014). In the present study, LSR lambs had a higher relative abundance of *Fibrobacteres* (3.87 vs. 0.99%), which aligns well with that of *Fibrobacter succinogenes* (2.42 vs. 0.66%). The abundances of *Fibrobacteres* and *Fibrobacter succinogenes* decline under SARA conditions (Fernando et al., 2010; Petri et al., 2013). In summary, the abundance of pH-sensitive bacteria (*Firmicutes, Fibrobacteres*, and *Bacteroidetes*) in rumen fluid were different at phylum level in the LSR and HSR lambs, suggesting that the dynamic ruminal pH is indeed lower in HSR lambs than LSR lambs when they were fed an identical diet.

The abundances of *R. albus, R. flavefaciens, Ruminococcus* 1 and *Ruminococcaceae* UCG-010 were lower in HSR lambs (2.27 vs. 1.11%, *P* < 0.001). However, the abundances of *Saccharofermentans, Ruminococcaceae* UCG-014 and *Ruminococcaceae* NK4A214 groups were higher in HSR lambs than in LSR lambs, indicating that the genera with in the family *Ruminococcaceae* have different pH sensitivities. Mao et al. (2013) observed an increase in *Ruminococcus* abundance (1.41% vs. 4.94%, *P* = 0.013) during grain induced SARA. Hook et al. (2011) suggested that *Ruminococcaceae* species can ferment fiber as well as starch and that their abundance increased in high-grain diets, but when SARA becomes severe, *Ruminococcus* abundance declines. It is possible that HSR lambs experience severe SARA that reduced the abundance of *Ruminococcus* in our study.

Despite the difference in the severity of acidosis between the LSR and HSR groups, the abundance of *Prevotella* 1 in the two groups was identical. This result was agreed with the qRT-PCR quantitation results of *Prevotella brevis* in this study. Mohammed et al. (2012) evaluated the severity of SARA during prepartum and postpartum periods and found that the abundance of *Prevotella* was not affected by the risk of SARA. In contrast, McCann et al. (2016) reported an increase in the abundance of *Prevotella* (rumen solid fraction) in the SARA-susceptible cattle. Petri et al. (2013) also found that the abundance of *Prevotella* positively correlated with the area below the pH threshold of 5.20 in heifers. The lack of difference in the abundance of *Prevotella* in the current study, compared to the McCann et al. (2016) study, could be attributed to the different SARA induction methods that was used. McCann et al. (2016) induced SARA by imposing a 50% feed restriction for 4 d and following that with *ad libitum* access to feed for 5 days, whereas the different SARA severity in the current study was caused by feeding lambs the same diet for 50 days. When SARA was induced by abruptly increasing the fermentable carbohydrate intake, the changes in ruminal fermentation were more acute than when the identical diet was provided during the experimental period in the current study. Furthermore, ruminal microflora that adapt to a new diet may need a long time (usually longer than 21 days) to became homeostatic, so 5 days may not have been long enough to allow *Prevotella* to adapt to the diet in the study of McCann et al. (2016); in contrast, microflora were given 50 days to adapt in our study. Differences in treatment method and adaption length of diet could account for the discrepant *Prevotella* in these studies.

The abundance of ruminal lactic acid producers *Lactobacillus* and *Lactococcus* were lower than 0.01% and have did not differ between LSR and HSR lambs in the present study. *S. bovis* is also an important lactate producer whose abundance increased under lactic acidosis conditions (Khafipour et al., 2009; Plaizier et al., 2017). The abundances of *Streptococcus bovis* and *Selenomonas ruminantium* were 3.91 and 7.26 fold greater in LSR lambs than in HSR lambs, indicating a level of synchrony between lactate utilizers and producers. These results agreed with those of McCann et al. (2016), who found that an increase in *Streptococcus bovis* is matched by increase in *Megasphaera elsdenii* abundance before and after grain-induced SARA in dairy cow.

Variation in susceptibility to acidosis or ruminal pH in ruminants mainly attributed to differences in their feeding behaviors (feed intake, chewing activities and sorting behavior) (Nasrollahi et al., 2017), epithelium VFA absorptivities (Gao and Oba, 2016), and rumen content passage rates when they intake the same diet because these factors affect the production and removal of VFA and protons in rumen. In the current study, feed was prepared in the form of TMR pellets that avoided sorting behavior by the animal. It can therefore be speculated that feeding behavior and saliva production between the LSR and HSR lambs were not different because of the identical DMI and peNDF intake. Although the VFA absorption and rumen content passage rates were not determined in our study, Gao and Oba (2016) showed that VFA absorption and rumen liquid passage rates cannot account for the differences in rumen pH between the cows with lower and greater SARA risk. Ruminal microflora are the connection between the feed (substrate) and degradation products (SCFA and NH_3_-N) that affect the ruminal dynamic pH. In turn, dynamic pH also affects bacterial communities which are sensitive to acidity. It is proposed that the different bacteria community between the LSR and HSR lambs were not only due to different risk of SARA, but also variation of SARA among the herd of lambs.

In our study, the amount of the four cellulolytic bacteria species were measured by qRT-PCR method. However, cautions must be exercised when comparing the qRT-PCR bacteria amount data to the results obtained by cultured-based approach. Bacteria amount determined by qRT-PCR cannot reflect the real activity of these species in the rumen because DNA may originate from inactive or dead cells that are not involve fiber degradation (Mosoni et al., 2007; Lettat and Benchaar, 2013). New approach such as RNA-based (Li et al., 2016) or culture-based (Mosoni et al., 2007) methods need to validate the results of the present work in further.

## Conclusion

This study investigated the correlation between the 2–4 h post-feeding ruminal pH and the amount of four cellulolytic bacteria in rumen when the lambs fed a comment diet. The abundances of the four pH-sensitive cellulolytic bacteria were uncorrelated with the 2–4 h post-feeding ruminal pH when they intake a common diet. These results indicated 2–4 h post-feeding ruminal pH may not be a suitable indicator of SARA risk in lamb herd. We adopted the amount of the four pH-sensitive cellulolytic bacteria as biomarker of SARA risk. Although all lambs were fed a common diet, lambs with fewer number of the four ruminal cellulolytic bacteria showed several symptoms of SARA, included lower ruminal pH and acetate to propionate ratio, higher concentration of lactate and proportion of propionate. Ruminal bacterial richness in the HSR was lower in HSR lambs than LSR lambs. The bacterial community in LSR lambs also clustered differently than that of HSR lambs. More specifically, HSR lambs had more *Firmicutes* organisms and fewer *Bacteroidetes* and *Fibrobacteres* organisms, suggesting that the species in these phyla were sensitive to the low-pH environment and independent of substrate composition in the present study.

## Author Contributions

FeL designed the experiment and prepared the manuscript. FaL designed the experiment and reared the animal. ZW, CD and XW analyzed the sample. WW and FM collected the samples. ZY unloaded the data to NCBI.

## Conflict of Interest Statement

The authors declare that the research was conducted in the absence of any commercial or financial relationships that could be construed as a potential conflict of interest.

## Footnotes

1 www.ccb.jhu.edu/software/FLASH/

2 www.drive5.com
