# Supplementary material for: Rumen Bacteria Communities and Performances of Fattening Lambs with a Lower or Greater Subacute Ruminal Acidosis Risk
Source: Front Microbiol. 2017 Dec 12;8:2506. doi: 10.3389/fmicb.2017.02506 (PMC5733016; doi:10.3389/fmicb.2017.02506)
Supplement: Supplementary file 1 [file Table_1.DOCX]

Table S1. Ingredients and chemical composition of the diets

| Item | Experimental diet |
| --- | --- |
| Ingredient，% of DM |  |
| Barley straw | 27.0 |
| Corn | 44.0 |
| Beet molasses | 5.0 |
| Soybean meal | 2.2 |
| Rapeseed meal | 4.5 |
| Cottonseed meal | 7.0 |
| Barley roots | 6.0 |
| Urea | 1.0 |
| Premix | 0.5 |
| Limestone | 1.3 |
| NaCl | 0.5 |
| NaHCO_3_ | 1.0 |
| Chemical composition, % of DM |  |
| CP | 15.7 |
| NDF | 36.0 |
| Ca | 0.63 |
| P | 0.30 |
| Starch | 28.5 |
