# Supplementary material for: Rumen Bacteria Communities and Performances of Fattening Lambs with a Lower or Greater Subacute Ruminal Acidosis Risk
Source: Front Microbiol. 2017 Dec 12;8:2506. doi: 10.3389/fmicb.2017.02506 (PMC5733016; doi:10.3389/fmicb.2017.02506)
Supplement: Supplementary file 2 [file Table_2.DOCX]

Table S2. PCR primers for amplifying target populations.

| Target species | Forward primer | Reverse primer |
| --- | --- | --- |
| General bacteria | CGGCAACGAGCGCAACCC | CCATTGTAGCACGTGTGTAGCC |
| *Fibrobacter succinogenes* | GGTATGGGATGAGCTTGC | GCCTGCCCCTGAACTATC |
| *Ruminococcus flavefaciens* | TCTGGAAACGGATGGTA | CCTTTAAGACAGGAGTTTACAA |
| *Ruminococcus albus* | CCCTAAAAGCAGTCTTAGTTCG | CCTCCTTGCGGTTAGAAC |
| *Butyrivibrio fibrisolvens* | GCCTCAGCGTCAGTAATCG | GGAGCGTAGGCGGTTTTAC |
| *Prevotella brevis* | GGTTTCCTTGAGTGTATTCGACGTC | CTTTCGCTTGGCCGCTG |
| *Streptococcus boivs* | TTCCTAGAGATAGGAAGTTTCTTCGG | ATGATGGCAACTAACAATAGGGGT |
| *Selenomonas ruminantium* | CAATAAGCATTCCGCCTGGG | TTCACTCAATGTCAAGCCCTGG |
